# Protein Based Nanostructures for Drug Delivery

**DOI:** 10.1155/2018/9285854

**Published:** 2018-05-16

**Authors:** Deepali Verma, Neha Gulati, Shreya Kaul, Siddhartha Mukherjee, Upendra Nagaich

**Affiliations:** Department of Pharmaceutics, Amity Institute of Pharmacy, Amity University, Noida, Uttar Pradesh 201301, India

## Abstract

The key role of protein based nanostructures has recently revolutionized the nanomedicine era. Protein nanoparticles have turned out to be the major grounds for the transformation of different properties of many conventional materials by virtue of their size and greater surface area which instigates them to be more reactive to some other molecules. Protein nanoparticles have better biocompatibilities and biodegradability and also have the possibilities for surface modifications. These nanostructures can be synthesized by using protein like albumin, gelatin, whey protein, gliadin, legumin, elastin, zein, soy protein, and milk protein. The techniques for their fabrication include emulsification, desolvation, complex coacervation, and electrospray. The characterization parameters of protein nanoparticles comprise particle size, particle morphology, surface charge, drug loading, determination of drug entrapment, and particle structure and in vitro drug release. A plethora of protein nanoparticles applications via different routes of administration are explored and reported by eminent researchers which are highlighted in the present review along with the patents granted for protein nanoparticles as drug delivery carriers.

## 1. Introduction

In recent years, there has been a drastic increase in research, technological development at atomic, and molecular and macromolecular scales which lead to the controlled manipulation and study of structure ranges from 1 to 100 nm. Nanoparticles fit into the category of colloidal drug delivery system which behaves like a whole unit with respect to its properties and transport mechanism. These are used as drug carrier system to improve the cellular uptake as well as body distribution. Nanoparticles became the major reason for the change in different properties of many conventional materials by virtue of their greater surface area per weight than microparticles which makes them to be more active drug carriers. Several types of nanoparticulate system which includes polymeric nanoparticles, polymeric micelles, solid nanoparticles, lipid based nanoparticles, for example, solid lipid nanoparticles (SLN), nanostructured lipid carriers (NLC), and lipid drug conjugate (LDC), liposomes, inorganic nanoparticles, dendrimers, magnetic nanoparticles, nanocrystals, and nanotubes. Various types of materials are used to prepare the nanoparticles which include polymers, lipids, polysaccharides, and proteins. There are some criteria for selection of matrix material for nanoparticles which are size of the nanoparticle, desired drug release profile, properties of the drug such as solubility and stability, and nature of the material, that is, biodegradability and toxicity. Recently, biopolymer based nanoparticles including protein nanoparticles are actively used in pharmaceuticals and nutraceuticals due to their low toxicity and biodegradability [1, 2].

Proteins are kind of natural molecules that show unique functionalities and properties in biological materials and manufacturing field. There are numerous nanomaterials which are derived from protein, albumin, and gelatin. These nanoparticles have promising properties like biodegradability, nonantigenicity, metabolizable, surface modifier, greater stability during* in vivo *during storage, and being relatively easy to prepare and monitor the size of the particles. These particles have the ability to attach covalently with drug and ligands [3–5]. Protein nanoparticles can be used in various targeted therapies, namely, pulmonary delivery, cancer therapy, tumor therapy, and vaccines, in which protein nanoparticles can be incorporated into biodegradable polymer in the form of microspheres for controlled and sustained release. The major aim in designing nanoparticle as a drug delivery system is to control particle size, surface area, and surface properties so that the nanoparticles carrying required amount of drug show desired pharmacological activity by releasing actives in order to achieve site-specific action. Proteins nanoparticles have certain unique functionalities and potential applications in both biomedical and material sciences [6]. They are recommended as ideal material for the preparation of nanoparticles because of their amphiphilicity which allow the nanoparticles to interact with both the drug and solvent. Nanoparticles derived from natural proteins are biodegradable, metabolizable, and easily adaptable to surface modifications to allow attachment of drug and targeting ligands [7]. They can be synthesized from various protein including water soluble proteins (e.g., bovine and human serum albumin) and insoluble protein (e.g., zein and gliadin). The comparison between synthetic and protein nanoparticles was discussed in Table 1.

**Figure 1:**
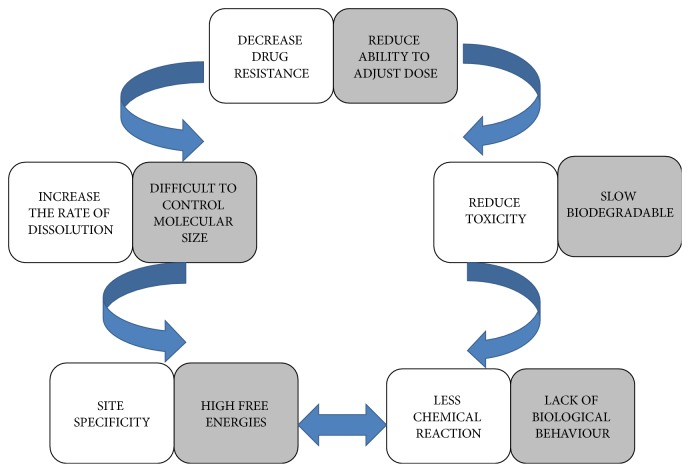
Pictorial presentation of positive aspects and negative aspects of protein nanoparticles.

Protein nanoparticles have plethora of advantages; namely, they help in reducing toxicity, enhance the release of drug, improve bioavailability, and provide better formulation opportunities. Protein nanoparticles are able to show better action at minimum dose and also decrease the drug resistance in body. Furthermore, the rate of dissolution and surface area of drug embed in the nanoparticle can also be enhanced. Nanoparticles can be delivered via using various routes of administration, including oral administration, vascular administration, and inhalation [49]. Important factor is to protect and preserve the drug while incorporated into the system without any chemical reaction. For this, many approaches are discovered; namely, modulation can be made into the choice of matrix constitution, site specificity can be achieved by attaching targeting legends to surface of particles, the system can be used for various route of administration, and the nanoparticles can improve the solubility and stability of encapsulated drugs. As the rule of nature, each and everything in universe has some positive and negative aspects. These are some problems associated with protein nanoparticles as particles can interact with living organism causing toxicity to depend upon chemical composition and the molecular size of particles becomes uncontrollable which can further affect the delivery of the system [50]. There is basic lack of understanding of the biological behavior of nanoparticles in terms of distribution in vivo both at the organ and at the cellular level [34]. Nanoparticles have high free energies due to their sizes. This causes aggregation and agglomeration. Nanoparticles are slow biodegradable which might cause systemic toxicity and reduce the ability to adjust dose [51]. Several positive and negative aspects of protein nanoparticles are discussed in Figure 1.

## 2. Excipients Utilized for the Synthesis of Protein Nanoparticles

**Figure 2:**
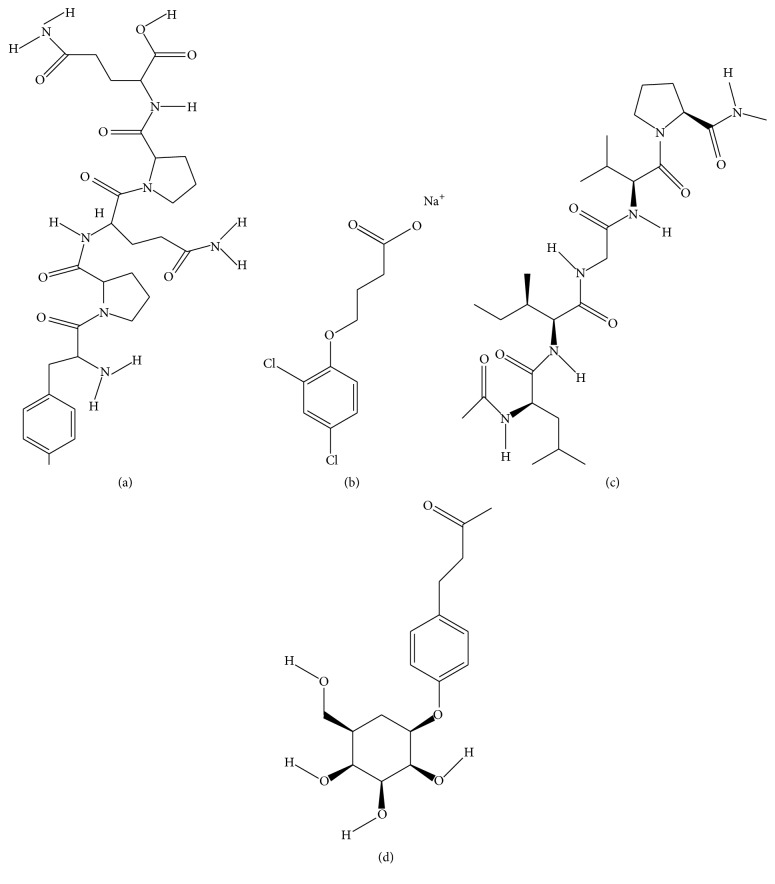
Chemical structure of (a) gliadin, (b) legumin, (c) elastin, and (d) zein.

These are plethora of excipients utilized for the preparation of protein nanoparticles. Many are extracted from natural sources, thus, making them suitable for drug delivery. For a quick look of excipients, a compilation is done in Table 2. The chemical structure of the proteins is given in Figure 2.

### 2.1. Albumin

Albumin is an attractive macromolecule carrier which can be obtained from a variety of sources including egg white (ovalbumin), bovine serum albumin, and human serum albumin (HSA). It is a water soluble protein which is utilized in maintaining the osmotic pressure, binding, and transport of nutrients to the cells. It is also soluble in diluted salt solution and has high solubility (up to 40% w/v) at pH 7.4. It can be heated up to 60°C up to 10 hours without showing any kind of denaturation effects [52–54]. Albumin is widely used in the preparation of nanospheres and nanocapsules. These albumin nanocarriers are nontoxic, are biodegradable, are easy to prepare, are nonimmunogenic, have well defined sizes, and also carry some reactive group (thiol, amines, and carboxyl). These group can work as ligand binding unit or surface modifier. The advantage of ligand binding albumin is that it can be easily attached by covalent linkage. The drug entrapped in the albumin nanoparticle can be easily digested by the enzyme protease. Lomis et al. (2016) investigate the effect of human serum albumin nanoparticles for cancer drug delivery [17]. A polymer based nanoparticles were formulated by using bovine serum albumin with the desolvation technique. The purpose was to increase drug aqueous solubility, sustained release, and targeted therapy in breast cancer. Ex vivo studies suggested that the albumin loaded nanoparticles help to improve drug release, enhance bioavailability, increase pharmacokinetics properties, and improve tissue target ability of drug. Noorani et al. (2015) formulated an albumin nanoparticle which reveals increase in an anticancer efficacy of albendazole in ovarian cancer xenograft model [18]. In this study, albendazole loaded bovine serum albumin nanoparticles was formulated with particle size ranges between 7 and 10 nm. In vitro cell proliferation studies show the highest killing efficacy of ovarian cancerous cell having least toxicity. Jithan et al. (2011) prepared albumin nanoparticles encapsulating curcumin intended for the treatment of breast cancer [19]. The study focuses on the development of human serum albumin nanoparticles containing paclitaxel by emulsion-solvent evaporation method. The yield of the nanoparticles was nearly 93% (w/w) and encapsulation efficiency of 82% (w/w). In vitro drug release and cytotoxicity assay were done on human breast cancer cell line (MCF-7) which displayed significant results.

### 2.2. Gelatin

Gelatin is most ancient proteinaceous material which is used for the formulation of nanoparticles. It is derived from controlled hydrolysis of fibrous, insoluble protein and collagen which is obtained from the skin, bones, and connective tissues. Gelatin is considered to be the biodegradable base for any nanoparticulate formulations. Gelatin is biodegradable, nontoxic, and easy to crosslink [55]. Due to some ionizable group such as amino, phenol, guanidine, and imidazole, it was emerged as potential system for the preparation of colloidal drug delivery system. It has another significant advantage that it can be sterilized, inexpensive, nontoxic, and noncontaminated with pyrogens and has relatively low antigenicity. The negative aspect of formulations consisting gelatin experienced that the outer layer of gelatin can cause crosslinking of intermolecular and intramolecular with respect to time, temperature, and humidity [56, 57]. To overcome this problem, some chemical crosslinkers like glutaraldehyde were added to the formulation which gives stability and shape and also enhance the circulation time for in vivo studies. There are two types of gelatin: A and B. Both gelatin can be produced by either acid or base hydrolysis; this leads to change in different properties of these two such as molecular weight, pH, viscosity, amino acid composition, and isoelectric points; namely, gelatin type A has pH 7–9 while gelatin type B has pH 4-5. Gelatin is approved by the United States Food Drug Administration (USFDA) as generally recognized as safe (GRAS) excipient used in pharmaceutical. Solanki et al. prepare gelatin nanoparticles as a delivery system for proteins [20]. The study shows the formulation of biodegradable hydrophilic gelatin nanoparticles by using protein, bovine serum albumin, and human serum albumin (BSA/HSA) with the help of precipitation method. The encapsulation efficiency for BSA and HSA was found to be 90% (w/w) and 80% (w/w), respectively. The controlled release of the protein was obtained 6 days in case of BSA and linear release was observed within 6 days in case of HSA.

### 2.3. Gliadin and Legumin

Gliadin is a gluten protein extracted from wheat. Gliadin appears to be suitable polymer for the oral and topical drug delivery system. Gliadin is generally used for the preparation of mucoadhesive formulation because it has capability to adhere on the mucus membrane. Its excellent properties, namely, biocompatibility, biodegradability, natural occurrence, nontoxicity, and stability, make it a suitable candidate as drug delivery system. Its hydrophobic nature and solubility permit the nanoparticles to protect the loaded drug for its controlled and sustained release [58, 59]. Gliadin exhibits great tropism, that is, indicative growth towards upper gastrointestinal regions, and has shown less presence towards intestinal region. Gliadin is rich in neutral and lipophilic amino acids residues whereas neutral amino acids promote hydrogen bonding with mucus membrane and lipophilic amino acids interact with biological tissues via hydrophobic interactions. Solanki et al. (2015) and Gulfam et al. (2012) formulated gliadin nanoparticles loaded anticancer drug and conducted in vivo studies by inducing apoptosis in the breast cells [21]. The study focuses on the formulation of gliadin and gliadin-gelatin composite nanoparticles for the controlled release of an anticancer drug, namely, cyclophosphamide by using electrospray deposition system. Gliadin nanoparticles containing cyclophosphamide show release in 48 h. In contrast, the gliadin-gelatin consisting nanoparticles show rapid manner of release. The demonstration for culture of breast cancer cells loaded with cyclophosphamide-7% gliadin nanoparticles was done for 24 hrs, resulting in apoptosis of cells. The confirmation for apoptosis of breast cancer cells was done by using western blotting method. Legumin also contains proteins in the pea seeds (*Pisum sativum*). It is an aluminous substance which shows similar function as casein as it consists of sulfur containing amino acids in seed meals. After aggregation and chemical crosslinking with glutaraldehyde, the protein molecule has the ability to form nanoparticles [60]. Mirshahi et al. (2002) shows development of protein nanoparticles from pea seeds after aggregation and chemical cross-linking with glutaraldehyde to study the adaptive immune response of legumin nanoparticles in rats [22]. The study focuses on the preparation of legumin nanoparticles by using pH-coacervation method and chemical crosslinking with glutaraldehyde. The purpose of the study is to avoid the use of organic solvents while obtaining the good yield, size, and surface charge.

### 2.4. Elastin

Elastin is an essential component in connective tissue that allows maintaining the elasticity and tensile strength of the tissues. The crosslinking in elastin occurs by two polyfunctional amino acids, that is, desmosine and isodesmosine. They are formed by oxidative deamination of three out of every four lysine side chains. So, the elastin was formed through lysine mediated crosslinking with its soluble precursor tropoelastin [61]. There are two types of elastin derived polypeptides that have been used for drug delivery system; namely, (a) *α* –o elastin undergoes aggregation under selective conditions of concentrations and temperature called cloud point, when the temperature is raised above cloud point, then elastin starting forming complex and (b) elastin-like polypeptides are repetitive peptide polymers sequences; these polypeptides are derived from tropoelastin and undergo an inverse phase transition which promote temperature dependent aggregation. The elastin-like polypeptides are highly soluble [62]. Mc Daniel et al. (2009) fabricated elastin-like polypeptide nanoparticles for drug delivery by electrospraying [23]. The study focuses on the formulation of bioresponsive elastin-like polypeptides (ELPs) nanoparticles by using electrospray. ELPs and drug are dissolved in organic solvent and the findings show that the particle diameter, polydispersity, and surface charge are showing significant results. The study suggests that the electrospray is an effective and flexible technique for the preparation of stimuli-responsive drug particle.

### 2.5. Zein

Zein consists of rich prolamine protein that contains hydrophobic amino acids, proline, and glutamine. Zein is highly used for films and coatings. Zein was approved by FDA as generally recognized as safe (GRAS) polymer for human application. Nanoparticles formed from zein proteins have been prepared to encapsulate several drugs and bioactive compounds including coumarin and 5-fluorouracil. In vitro release of coumarin was reported to be over 9 days from zein [63]. Zargar et al. (2016) investigate zein bionanoparticles as a novel green nanopolymer dispersive solid-phase extraction adsorbent for separating and determining trace amounts of azorubine in different foodstuffs [24]. The formulation of zein nanoparticles was done by using dispersive solid-phase extraction (DSPE). The present study shows the analysis of four different food samples (soft drink, pastel gummies, ice cream, and smarties) and obtains the azorubine (AZ) range of 94.6% to 103.2%. The method shows convenient and fast method for calculating the AZ value in food sample. Dhanya and Haridas (2012) develop zein-pectin nanoparticle as drug carrier [25]. The aim of the study is to formulate biodegradable and nontoxic zein-pectin nanoparticle using ultrasonication method. Nanoparticle consists of a hydrophobic zein core and a pectin shell which is hydrophilic in nature. Chen (2012) fabricated zein nanoparticle-biopolymer complexes to deliver essential oils in aqueous dispersions [26]. The study shows the formulation of zein nanoparticles while dispersing the zein solution with polar solvent in the water. To stabilize zein nanoparticles, sodium caseinate was used by dispersing it in hot 50% (v/v) aqueous ethanol solution with polymers in water.

### 2.6. Soy Protein

Soybean (glycine max) is the most abundant source of plant protein. The enriched form of soy protein is known as soy protein isolates which is reported to be of highly nutritional values and ingredients functionalities. The important component for soy protein isolate is glycinin and *β* conglycinin [64, 65]. Upon addition of crosslinking agents soy protein isolate forms aggregate and at certain temperature microspheres, hydrogels, and polymer blends were formed. Soy protein nanoparticles can be prepared by addition of either desolvation agents or glycinin fraction of defatted soy flour extraction using simple coacervation method. Teng et al. (2012) synthesized nanoparticle from soy protein and explored its application for nutraceutical encapsulation [27]. The study shows the formulation of soy protein nanoparticles by using dispersion, desolvation, drug incorporation, crosslinking, and evaporation. Curcumin was used as an active ingredient to be incorporated into the nanoparticles. The average size of the nanoparticles was 220.1–286.7 nm and also achieved the highest encapsulation efficiency of 97.2%. Liu and Tang (2013) prepare soy protein nanoparticle aggregates as pickering stabilizers for oil-in-water emulsions [28]. The study investigates the formulation of soy protein nanoparticles which was used as pickering stabilizer for oil-in-water emulsion. The finding shows that heated soy protein isolate shows more significant results in comparison to unheated one. The droplet size also gets decreased but stability against coalescence and creaming will enhance which lead to formation of gel-like network that could entrap the oil droplets. Hence, the soy protein emulsion will help to stabilize the pickering and is used in nutraceuticals.

### 2.7. Milk Protein

Milk consists of several kinds of protein with different function and properties. The use of milk protein as drug delivery vehicles is a new trend that has received much attention [66, 67]. There are two milk proteins which were used in drug delivery application, namely, *β*-lacto globulin (BLG) and casein. The BLG consists of two disulphide bonds and one free thiol group. BLG has good gelling ability which was used as drug delivery application. Another milk protein is casein which exists as micelles size of ranges of 100–200 nm; it is generally used for the transportation of calcium and amino acid; micelles of calcium have no fixed structure why the change in temperature, pH, ionic strength, and water activity casein can withstand heat and mechanical forces. Huang et al. (2015) prepared milk protein coated magnetic nanoparticle enabling oral drug delivery with high stability in stomach and enzyme-responsive release in small intestine [29]. The study focuses on the formulation of layer by layer milk protein casein coated iron oxide nanoparticles. Doxorubicin (DOX) and indocyanine (ICG) were selected as the drugs which were incorporated into the inner layer and subsequently coated with casein. With the help of casein coating, the drug release rate was increased in simulated intestine condition.* Ex vivo* studies show that the layer by layer milk protein loaded with doxorubicin and indocyanine improves the absorption in small intestine sacs and has the capability to cross the microvilli. In vivo studies were also conducted by oral administration of nanoparticles and checked by imaging which indicates no significant degradation of stomach and then gets accumulated in small intestine.

## 3. Formulation Techniques and Evaluation Parameters of Protein Nanoparticle

A large number of macromolecules were used in the preparation of nanoparticles. The macromolecules are albumin, gelatin, and legumin. These macromolecules are extensively used because of their natural properties including biodegradable and biocompatibility. During the formulation of protein nanoparticles there will be conformational change, namely, composition and concentration, and the chemical changes, namely, pH, ionic strength, temperature, and type of solvent of protein occurring. Usually to stabilize these conditions, surfactants are used [68, 69]. Comparative procedures are given in Table 3.

### 3.1. Emulsification Method

**Figure 3:**
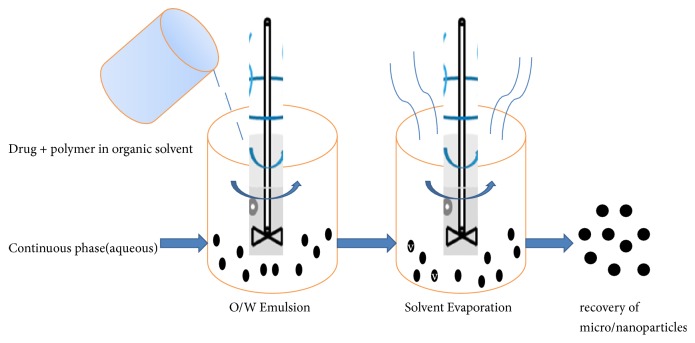
Diagrammatic representation of emulsification technique for protein nanoparticles formulation.

Initially, this method was first discovered by Scheffel and his coworkers in 1972 to prepare albumin spheres but his work was reconducted by Gao and his coworkers in 1995. In this method, an aqueous phase of albumin was prepared with distilled water and organic phase plant oil such as cotton seed oil [53, 70]. Now the oil and water phase was mixed in the container under mechanical homogenizer until an oil-water (o/w) emlusion was perpared. The above emulsion will be added into the preheat oil over 120°C drop by drop. Now there will be evaporation of water and irreversible destruction of albumin which lead to formation of nanoparticles. The resulting particles were suspended into cold ice bath. In Figure 3, a diagrammatic representation of emulsification technique was given.

### 3.2. Desolvation Method

**Figure 4:**
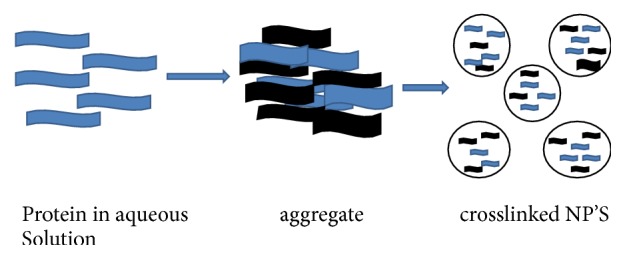
Flowchart presentation of desolvating/coacervation technique.

This method was given by Marty and his coworkers in 1978. This method is also called coacervation method. Under this method, a desolvation agent such as natural salt or alcohol was added into the aqueous solution of albumin [71, 72]. By adding of desolvation agents, protein starts changing its structure slowly. Now at certain level protein, clumps will be made and finally nanoparticles will be formed due to crosslinking. To separate the particles, the turbidity of the system should be increased. A flowchart presentation of protein nanoparticles by using desolvation/coacervation technique was discussed in Figure 4.

### 3.3. Complex Coacervation Method

**Figure 5:**
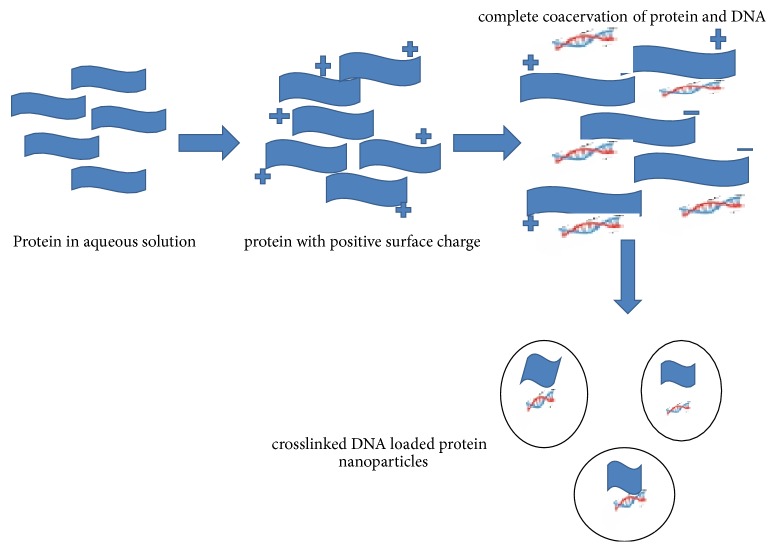
Pictorial presentation of complex coacervation technique for protein nanoparticles preparation.

This method is generally suited for the DNA entrapment. Since proteins are amphoteric in nature, they can be made cationic or anionic by adjusting the pH. In this method, proteins in aqueous solution were taken; then pH was adjusted due to the particles with positive charge coming upwards. Then, a mixture of DNA and salt solution was prepared and added into the above aqueous protein solution. By the interaction of DNA and protein complex, coacervation occurs. Simultaneously, addition of crosslinker such as 1-ethyl-3-(3-dimethylaminopropyl)carbodiimide (EDC) was done to obtain the crosslinked DNA loaded protein nanoparticles. In this last step DNA is physically entrapped in the protein matrix. There is an alternative method in which cationized protein was used to form complex with DNA [73, 74]. For this, a cationized gelatin was taken and attached covalently to cholamine. Firstly, gelatin nanoparticles were prepared by coacervation using acetone as desolvating agent followed by crosslinking agent such as glutaraldehyde. Then, cholamine was conjugated with surface of gelatin nanoparticles and DNA was absorbed. In Figure 5, the preparation of protein nanoparticles by using complex coacervation technique is shown.

### 3.4. Electrospray Technique

**Figure 6:**
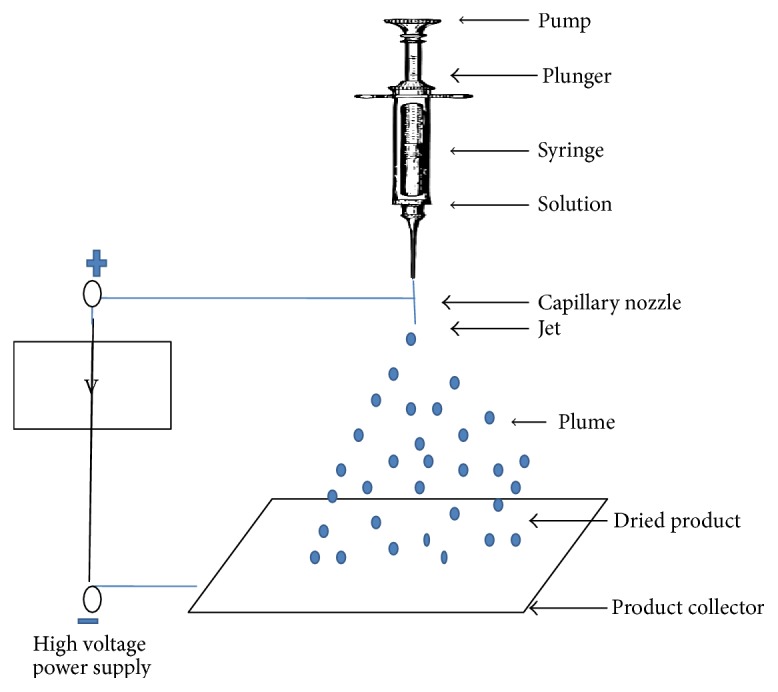
Preparation of protein nanoparticles by electrospray technique.

**Figure 7:**
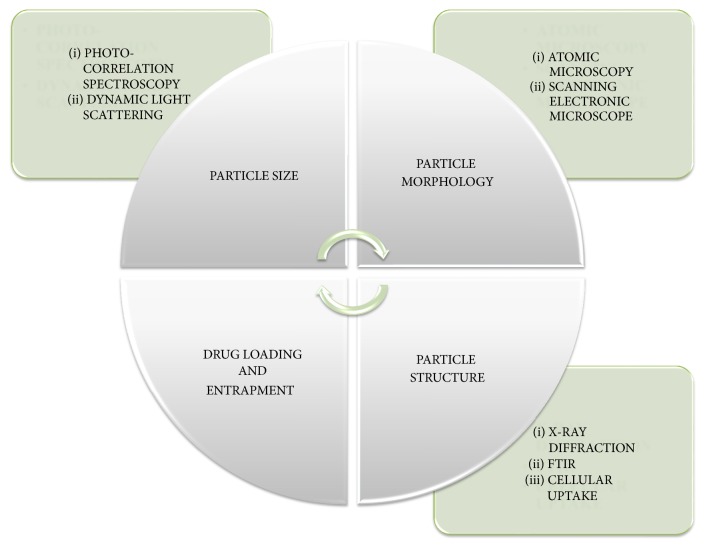
Pictorial presentation for characterization of protein nanoparticles.

Electrospray is a new technique used in the preparation of protein nanoparticles. This technique is generally used for gliadin and elastin peptide nanoparticles. In this method, high voltage is applied to the protein solution supplied through an emitter which emits a liquid jet stream through a nozzle which helps in forming an aerosolized size liquid consisting of drug and nucleic acid [75, 76]. The pictorial presentation of protein nanoparticles by using electrospray technique was given in Figure 6. The prepared protein nanoparticles are characterized by several parameters as mentioned in Table 4 and Figure 7.

## 4. Patents

A patent is defined as a legal document which grants exclusive rights to the inventor or provides some kind of monopoly over the invention. Over the last decade a large number of novel studies have been carried out by various researchers worldwide. Studies on synthesis of protein nanoparticles using varied sources, different techniques, and delivery carriers have also resulted in filing of patents around the globe. Some recent patents based on protein nanoparticles are discussed given in Table 5.

## 5. Biomedical Application of Protein Nanoparticles

### 5.1. Routes

**Figure 8:**
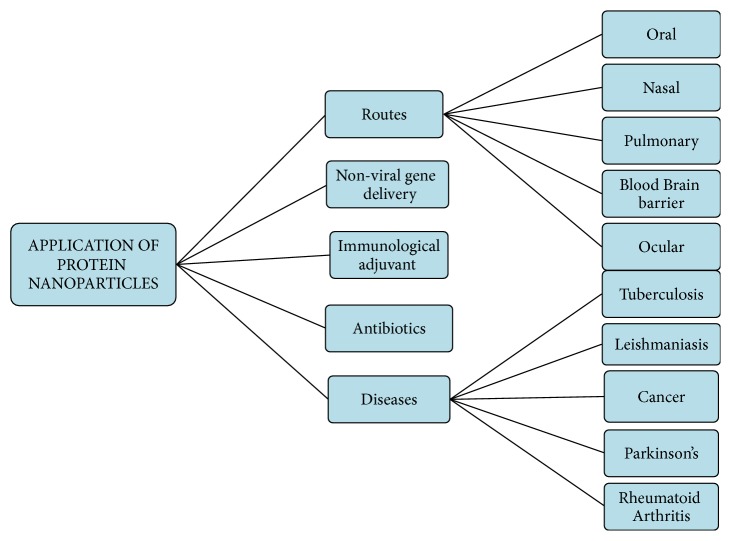
Application of protein nanoparticles.

There are different applications of protein nanoparticles as carriers for the delivery of proteins, drugs, and peptides via different routes of administration which have been discussed further. Different biomedical application of protein nanoparticles can be shown in Figure 8.

#### 5.1.1. Oral Route

Oral administration is the most preferred route for any kind of drug applications as this route shows different advantages like patient convenience and compliance, avoiding contaminations and infections [20, 21]. However, protein and peptides exhibit poor oral bioavailability due to lower permeation across intestinal epithelium, aggregation, and denaturation. Hindrance to oral administration of protein and peptide can be categorized as physical, chemical, and enzymatic barriers [22]. The physical barrier is attributed mainly to the continuous monolayer of intestinal epithelial cells which highly express intercellular tight junctions. Physicochemical properties of polymeric NPs can be optimized to facilitate transport across intestinal epithelial cells. There is plethora of protein oral delivery technologies under development by companies and some are discussed in Table 6.

#### 5.1.2. Nasal Route

Nasal route is commonly used for noninvasive protein and peptide delivery. Recent advancement in biotechnology, inhalation devices, and targeting motifs has considerably raised research interest in protein and peptide delivery via this route [22]. It offers advantages including large surface area, highly vascularized mucosa, porous endothelial membrane, lower enzymatic activity relative to GIT, and avoidance of first-pass metabolism. However, pattern of deposition and size distribution through delivery device and nasal clearance mechanisms might pose a significant challenge to protein delivery. Following intranasal administration, proteins can be absorbed directly into systemic circulation, into central nervous system (CNS), or across GIT. Miacalcin®, DDAVP®, Synarel®, Fortical®, and Syntocinon® are few of the marketed proteins and peptides for nasal administration [23].

#### 5.1.3. Pulmonary Route

**Figure 9:**
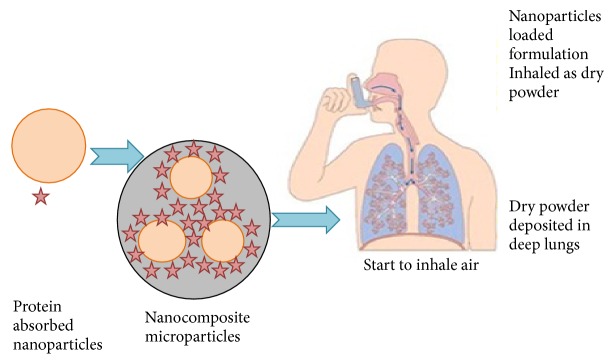
Schematic illustration of pulmonary route of nanoparticles.

Pulmonary route is one of the most commonly investigated noninvasive routes to improve absorption of proteins and peptides. This route provides numerous advantages including enormous absorptive surface area (100 m^2^), high vascularization, thin alveolar epithelial membrane (0.1–0.2 *μ*m), and low enzymatic activity despite these advantages; several factors may regulate pulmonary protein and peptide absorption. Alpha 1-antitrypsin-loaded PLGA NPs have been anticipated as a promising formulation for the treatment of respiratory diseases. In Figure 9, we have schematic representation of pulmonary route of nanoparticles.

#### 5.1.4. Blood Brain Barrier Route

**Figure 10:**
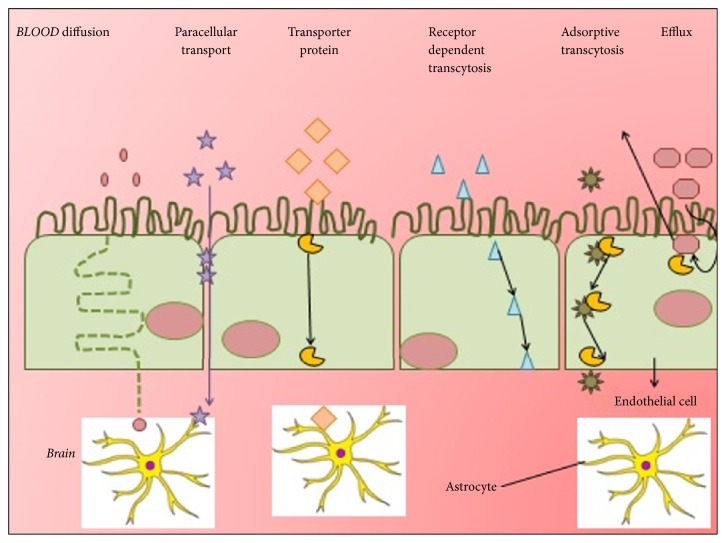
Various transport pathway through blood brain barrier.

Protein nanoparticles have the ability to cross blood brain barrier that cannot be crossed by normal drug through IV injection. The protein nanoparticle bound drugs include loperamide, tubocurarine, and doxorubicin. Different transport pathways of blood brain barrier can be shown in Figure 10.

#### 5.1.5. Ocular Therapy

Protein nanoparticles exhibit a considerably longer half-life in the eye than eye-drops. Pilocarpine bound to gelatin nanoparticles substantially prolonged the intraocular pressure reduction in rabbits with experimental glaucoma as well as the meiosis time in comparison to a pilocarpine eye-drop solution.

### 5.2. Nonviral Gene Delivery

Cationized gelatin nanoparticles have shown the potential of being a new effective carrier for nonviral gene delivery. The major benefit of gelatin nanoparticle is not only the very low cell toxicity, but also their simple production combined with low cost [29].

### 5.3. Immunological Adjuvant

Gelatin nanoparticles are used as immunological adjuvant to enhance both humoral and cellular responses to antigen. Sundar et al. (2010) used gelatin-DNA nanosphere coacervate as gene delivery vehicle to express the CFTR-gene into human tracheal epithelial cells [77].

### 5.4. Antibiotics

In antibiotics also protein nanoparticles show effective results. Antibiotics show an increase in efficacy or a decrease in toxicity after binding to protein nanoparticles. Amoxicillin and gliadin nanoparticles bearing amoxicillin (AGNP) both showed antihelicobacter pylori, but the required dose for complete eradication was less in AGNP than in amoxicillin [77, 78].

### 5.5. Diseases

According to different types of diseases the applications of protein nanoparticles have been further discussed below.

#### 5.5.1. Tuberculosis (TB)

TB is caused by bacteria called mycobacterium tuberculosis and if not treated on time can lead to mortality. Medication for Tb is available in market but nanotechnology comes as an emerging technology in this field because it provides the controlled release to the infected cells for an extended period of time via providing effective delivery system [79]. Rifampicin- (RIF-) loaded gelatin nanoparticles cause less harm to normal cells than free drug shown in cytotoxicity studies and increased the drug targetability by reducing its dose. RIF gelatin nanoparticles were found to be accumulated in various organs as compared to plain RIF. Also the RIF nanoparticles will lead to improving the pharmacokinetics of drug and it will provide sustained plasma level by enhancing the mean residence time and area under the curve [80]. Isoniazid loaded mannosylated gelatin nanoparticles show significant decrease in bacterial counts in the lungs and spleen of tuberculosis infected mice via intravenous administration and also reveal the reduction in hepatotoxicity of the drug and works as potent and safe carrier for management of tuberculosis [81].

#### 5.5.2. Leishmaniasis

Leishmaniasis is a microbial disease caused by protozoan parasite* Leishmania donovani*. Amphotericin B (AmB) is a polyene antibiotic which is used for the treatment of visceral leishmaniasis. AmB loaded functionalized gelatin nanoparticles reduce the cytotoxicity and enhance the uptake by macrophages [82]. In vitro and in vivo 1,2-diacyl-sn-glycero-3-phospho-1-serine coated gelatin nanoparticles loaded AmB shows high accumulation of AmB in liver and spleen which enhance antileishmanial activity [83].

#### 5.5.3. Cancer Therapy

Cancer is generally a group of diseases traditionally treated by chemotherapy and radiotherapy. However, there are many side effects in traditional therapy, namely, drug toxicity and drug resistance, to overcome the fact that nanoparticles are being explored for targeted drug delivery. Paclitaxel loaded gelatin nanoparticles show in vitro and in vivo drug solubility in rate limiting manner in aqueous medium. Intravesical paclitaxel gelatin nanoparticles showed favorable bladder tumor targeting and decrease in the systemic absorption. Pacitaxel loaded gelatin nanoparticles show constant drug release which overcomes the drug dilution problem and due to sustained drug level the treatment frequency may decrease [84]. Doxorubicin- (DOX-) polyethylenimine (PEI) loaded in human serum albumin (HSA) nanoparticles was found to be showing good results to treat the breast cancer. DOX-PEI-loaded HSA loaded observed less cytotoxicity and enhanced the biocompatibility of the formulation. Also in transfection conditions only 80% of cells were transfected with HSA nanoparticles contained tetramethylrhodamine- conjugated bovine serum albumin [85]. 

#### 5.5.4. Parkinson's Disease

Parkinson's disease (PD) is a chronic disorder of central nervous system which is primarily caused by loss of dopaminergic cells in the substantia nigra region which lead to resting tremor, muscular rigidity, bradykinesia, and postural instability [86, 87]. Neuropeptide substance P (SP) loaded gelatin nanoparticles (SP-GNP) reveal better cell viability and decrease the degree of apoptosis compared to normal SP solution, whereas the SP is a mediator of neuroimmunomodulatory activities and neurogenic inflammation in the central and peripheral nervous system [88].

#### 5.5.5. Rheumatoid Arthritis

Rheumatoid arthritis (RA) is an autoimmune disease which affects the joints of the body and causes disability and joint damage and decreases the quality of life. Methotrexate (MTX) is commonly used for the treatment of RA which helps to overcome the specificity related to inflamed tissues and increased the half-life. Meanwhile MTX-human serum albumin (HSA) showed decrease in synovial fibroblast and cartilage degradation [89].

## 6. Conclusion

The development of nanoparticles drug delivery system is accepted to play major role in treatment of various life-threatening diseases as it provides safe, effective, and stable therapeutics effects, whereas protein nanoparticles hold promising results in nasal, pulmonary, oral, and ocular delivery, nonviral gene delivery, blood brain barrier route, and immunological adjuvant. Also numerous protein synthesized nanoparticles like albumin and gelatin show advancement in commercial level. Recently new proteins are obtained from plants and milk can also be used in the nanotechnologies for the formulation of new drugs which shows some effective results. Advancement in various field of nanoparticles can lead to the enhancement in application of protein nanoparticles for the treatment of diseases, although the applications of protein nanoparticles for various diseases have already produced some exciting results and also hold some greater promise in future.

## Figures and Tables

**Table 1:** Comparison between Synthetic and Protein Nanoparticles.

S. number	Parameters	Synthetic Nanoparticles	Protein Nanoparticles	Ref. number
(1)	Definition	These are colloidal polymeric particles with a therapeutic agent either dispersed in the polymeric matrix or get encapsulated in the polymer.	These are kinds of natural molecules showing unique functionalities and properties in biological materials.	[4]

(2)	Size	10–100 nm	1–100 nm	[8, 9]

(3)	Polymers	Poly lactide (PLA) Poly lactide-co- glycolide (PLGA) Poly epsilon-caprolactone (PCL) Poly Isobutylecyanoacrylate(PIBCA) Poly acrylate (Eudragit)	Albumin Gelatin Gliadin Legumin Elastin Soybean Zein Milk protein	[10]

(4)	Formulation Technique	(i) Emulsification-solvent Diffusion (ii) Nanoprecipitation (iii) Emulsion evaporation (iv) Polymerization method (v) Double emulsion (vi) Salting out method	(I) Emulsification method (II) Desolvation method (III) Complex coacervation method (IV) Electro-spray method	[11]

(5)	Merits	(i) It will help to minimize the toxicity of drug towards specific site of delivery. (ii) It provides reduction in fluctuations in therapeutic ranges by improving the bioavailability. (iii) Improves the stability of the drug. (iv) Reduce dosing frequency. (v) Smaller particle size reduce potent irritant at site.	(i) Proteins are able to show better action at minimum dose. (ii) It will also helps to decrease the drug resistance in the body. (iii) The rate of dissolution and surface area embedded in nanoparticles can also be enhanced. (iv) There will be less chemical reaction occurs.	[12, 13]

(6)	Demerits	(i) Clustering of nanoparticle into bigger arrangement may lead to change in morphology of the drug. (ii) Rupturing of small particles in bulk can occur. (iii) Once enter into the body can be forbidden by any action or adverse effects.	(i) There will be difficulty in controlling its molecular size. (ii) The ability to adjust the dose will be reduced. (iii) Due to lack of biological behavior unable to identify the nature of nanoparticles. (iv) Nanoparticles posses high energies due to their size.	[14]

(7)	Applications	(i) For tumor targeting a concentrated dose of drug will be given which will lead to enhance permeability and retention effect. (ii) Provide better permeability towards blood brain barrier with specific receptor –mediated transport system. For ex- poly (butyl cyanoacrylate) nanoparticles was able to deliver hexapeptide, doxorubicin into the brain. (iii) Nanoparticles loaded with plasmid DNA will also serve efficient gene delivery.	(i) Protein nanoparticles provide a new option for the oral intake of peptides via nanostructure drug delivery. For instance, Insulin loaded nanoparticles can preserve the insulin activity and produce blood glucose reduction. (ii) Nasal route offers an advantage by enhancing the surface area and lower enzymatic activity relative to GIT. (iii) In case of antibiotics protein nanoparticles show significant results via decreasing the toxicity after protein binding. (iv) For ocular therapy it exhibits the longer half life by prolonging the intraocular pressure.	[15, 16]

**Table 2:** Compiled research investigations for numerous active loaded Protein Nanoparticles.

Proteins	Biological source	Properties and functions	Reference number
Albumin	Egg white (ovalbumin), bovine serum albumin and human serum albumin (HSA)	Albumin is a water soluble protein, nontoxic, biodegradable, easy to prepare, nonimmunogenic, easily attachable to covalent linkage. Prepared as nanospheres and nanocapsules.	[17–19]

Gelatin	Controlled hydrolysis of fibrous, insoluble protein and collagen which is obtained from the skin, bones and connective tissues.	Easy to crosslink, easily sterilized, inexpensive in nature, no contamination with pyrogens. Gelatin type A has ph 7–9 while gelatin type B has ph 4-5. Prepared as microspheres and nanoparticles.	[20]

Gliadin and legumin	Gliadin is a gluten protein extracted from wheat. Legumin also contains proteins in the pea seeds (*Pisum sativum*).	Biocompatibility, biodegradability, natural occurrence, nontoxic and stability, hydrophobic nature, and solubility. Gliadin is suitable polymer for the oral and topical drug delivery system. Used for the preparation of mucoadhesive formulation because it has capability to adhere on the mucus membrane.	[21, 22]

Elastin	Connective tissue	To maintain elasticity and tensile strength of the tissues. (1) *α* -elastin which undergo aggregation of conc. and temp. Called as cloud point, when raised form complex. (2) Polypeptide elastin is repetitive peptide polymers sequences, these derived from tropoelastin.	[23]

Zein	It consists of rich prolamine protein that contains hydrophobic amino acids, proline, and glutamine.	Nontoxic, stable, and biodegradable. Nanoparticles formed from zein proteins have been prepared to encapsulate several drugs and bioactive compounds including coumarin, 5-fluorouracil. Also used in highly used for films and coating.	[24–26]

Soybean (glycine max)	Obtained from plant sources in enriched from of soy protein.	The important component for soy protein isolate is glycinin and *β* conglycinin. Upon addition of cross linking agent's soy protein isolate form aggregate and at certain temperature microspheres, hydro gels and polymer blends were formed conglycinin.	[27, 28]

Milk Protein	Obtained from milk resources	There are two types of milk protein used in drug delivery application which are *β*-lacto globulin (BLG) and casein. The BLG consists of two disulphide bonds and one free thiol group. BLG has good gelling ability which was used as drug delivery application. Casein exist as micelles size of ranges of 100–200 nm, it used to transport the calcium and amino acid. Micelles of calcium have no fixed structure why the changes like temperature, pH, ionic strength, and water activity occur. Casein can withstand with heat and mechanical forces	[29]

**Table 3:** Summarized formulation techniques of protein nanoparticles.

Emulsification method	Desolvation method	Complex coacervation method	Electrospray technique
An aqueous phase of albumin mixed in distilled water ⇓ Organic oil phases in which plant oil such as cotton seed oil (room temp.) ⇓ Take oil and water phase and mixed under mechanical homogenizer until an o/w emulsion was prepared ⇓ The emulsion will be added into the preheat oil over 120°C drop by drop ⇓ Now the rapid evaporation of existing water and albumin irreversible destructive ⇓ The nanoparticles are formed and were suspended into cold ice bath.	Desolvation agent such as natural salt or alcohol was added into the aqueous solution of albumin ⇓ Addition of desolvation agents, protein starts changing its structure slowly ⇓ At certain level protein clumps will be made and finally nanoparticles will be formed due to crosslinking. (To separate particles the turbidity of the system should be increased.)	Protein in aqueous solution was taken and while adjusting the pH, the particles with positive charge comes upwards ⇓ Now a mixture of DNA and salt solution was prepared and added into the above protein aqueous solution ⇓ By the interaction of DNA and protein complex coacervation occurs ⇓ Addition cross linker such as 1-ethyl-3-(3-dimethylaminopropyl)carbodiimide (EDC) was done and cross linked DNA loaded protein nanoparticles was made. (In this last step DNA is physically entrapped in the protein matrix.)	In this method high voltage was applied to the protein solution supplied through an emitter which emits a liquid jet stream ⇓ Through a nozzle which helps to form an aerosolized size liquid consist of drug and nucleic acid ⇓ This technique is generally used for gliadin and elastin peptide nanoparticles

**Table 4:** Compiled evaluation parameters for the protein nanoparticles.

S. number	Parameter	Specification	Ref. number
(1)	Particle size	Due to their size and mobility nanoparticles have higher intracellular uptake as compared to microspheres. It was also reported that nanoparticles have the ability to cross blood brain barrier due to the opening of tight junctions by hyper osmotic pressure which helps to give sustained release of therapeutic agents.	[30, 31]

(1.1)	*Photon-correlation spectroscopy (PCS)*	In this method the time decay of the near particle caused by the Brownian motion which helps to evaluate nanoparticle via Strokes–Einstein relation and the interpretation of particle size is least ambiguous with a narrow distribution, an effective diameter and polydispersity index are measurable even with broad distributions. The major disadvantage of this technique is it does not produce a high-resolution histogram of the size distribution.	[32]

(1.2)	*Dynamic light scattering (DLS)*	This technique helps to observe the particle size of random pattern in suspension which compares larger particle size to smaller particle size.	[33, 34]

(2)	Particle Morphology	To observe the physicochemical properties which lead to revolutionize electronic, diagnostic, and therapeutic applications.	

(2.1)	*Atomic microscopy (AFM)*	This tool used for direct measurements of microstructural parameters and unraveling the intermolecular forces at nanoscale level with atomic-resolution characterization.	[35]

(2.2)	*Scanning electronic microscope (SEM)*	It helps to identify the signals that derive from electron-sample interactions reveal information about the sample including external morphology (texture), chemical composition, and crystalline structure and orientation of materials making up the sample. It has high resolution type of fractions, more than 1000 times better than the optical diffraction and particle surface was scanned under high energy beam.	[36, 37]

(3)	Surface charge	Zeta potential analysis is a technique for determining the surface charge of nanoparticles in solution (colloids) which possess a positive or negative electrostatic charge. Zeta potential also helps to understand the nanoparticle surface and predicting the long term stability of the nanoparticle.	[36]

(4)	Drug loading	Drug loading can be defined as the amount of drug bounded per mass of polymer usually in moles of drug per mg of polymer. Drug can be bound to nanoparticles either by the polymerization or adsorption.	[17, 18]

(5)	Determination of drug entrapement	Determined the UV-spectrophotometer or HPLC (*w*) absorbance. The amount of drug in supernatant was subtracted from the total amount of drug added during formulation (*W*). Effectively, (*W* − *w*) will be amount of drug entrapped in the nanoparticles- Drug entrapment (%) = (*W* − *w*)/*W* × 100.	[19]

(6)	Particle structure	To analyze the nature and modification in confirmation, folding, and chemical bonding.	

(6.1)	*X-ray diffraction*	The purpose of XRD is to investigate the structure of crystalline materials and also analyze their phase composition, crystallite size, shape, lattice, etc.	[38]

(6.2)	*Fourier transform infrared spectroscopy (FTIR)*	The principle of FTIR provides that a molecule is exposed to infrared rays absorbs infrared energy at frequencies which are characteristic to that molecule and provide information about the structural details of proteins in solution with greater spatial and temporal resolution.	[38]

(6.3)	*Cellular uptake*	Cellular uptake of nanoparticles is determined by tagging the nanoparticles with fluorescent tags followed by incubating these fluorescence-tagged nanoparticles with cells and their visualization under confocal laser scanning microscope.	[35]

**Table 5:** Various patents on protein nanoparticles.

Cited patent	Title & abstract	Ref. number
US8057839 B2	*Nanoparticulated whey proteins (Bovetto et al.)* The present invention is about the production of nanoparticulate whey protein by allowing a solution containing whey protein to a specific temperature, time and pH. The resultant whey protein formed was measured diameter of less than 1 *μ*m. Further on freeze–drying, the whey protein nanoparticles attain similar physical and morphological properties as in solution form. These nanoparticulates were found to have protein efficiency ratio of at least 100. These nanoparticulated whey protein used as emulsifiers, fat substitute, micellar casein substitute, whitening, foaming, and texturing agent.	[39]

US9233110	*Protein nanocarriers for topical delivery (Perumal et al.)* The present invention is based on the development of prolamine based nanoparticulate topical formulation of retinol and its compound. The developed formulation was used to cure the skin diseases including acne, psoriasis, keratinization disorders, skin discoloration and cutaneous malignancies. The diameter of the nanoparticle ranges about 75 nm–300 nm. Phospholipids and pluronics are also used as penetration enhancers. The nanoparticles loaded with retinoid provide less irritation to the human skin, also provide chemical stability, enhances therapeutic effect, low toxicity, and less skin irritation.	[40]

EP2625966A1	*Nanoparticles comprising a vegetable hydrophobic protein and a water miscible non-volatile organic solvent and uses there of (Salman et al.)* The current invention deals with the biocompatibility of nanoparticulate delivery system consisting nanoparticle based on vegetables hydrophobic proteins, involves zein and non-volatile organic solvents. This method shows high encapsulation efficiency for both hydrophilic and hydrophobic molecules, and allows the successful coating of nanoparticles with polyanions.	[41]

US20160220502 A1	*PotatoProtein Nanoparticles (Livney)* This invention focus on the formulation of potato protein loaded nanoparticles, a bioactive compound having potato protein and beverages or food supplemented with the nanoparticles. For the formulation of nanoparticle loaded with potato proteins and bioactive compounds comprising in two different mixture and mix them with intensive stirring and dried them. The bioactive compound of potato protein contains an oil soluble vitamin, a polyunsaturated fatty acid, an antioxidant and omega-3-fatty acid.These bioactive compounds contains maximal aqueous solubility below 1 g/l. In other part the clear solution of bioactive ecopoumd can be made with the addition of emulsifier to make clear beverage.	[42]

US20160095917	*Dps Fusion Proteins For Use In Vaccines And Diagnostics (Ni)* The present invention provides information regarding diagnosis, treatment and prevention of diseases by using Dps fusion proteins. Dps fusion proteins consisting at least one protein or peptide fused to Dps. Dps fused protein is soluble in spite of its poor solubility. Because of its thermo stability it has the capability to withstand at high temperature and also enhance immune responses.	[43]

US9,271,943	*Arsenic compound solution and albumin nanoparticle and lyophilized preparation entrapping arsenic compound (Lou et al.)* The invention based on the preparation of albumin nanoparticles loaded with arsenic solution and same nanoparticles were prepared by lyophilization method. The solution of arsenic compound was prepared by using arsenic powder in sterile deionized water and dripping it into NaOH solution to obtain pH 7.5–9. This mixture was dissolved in albumin solution in equal proportion. Freeze drying follws the same procedure by addition of cryoprotectant in combination of mannitol, glucose, sucrose at temperature −80 and 40C. Albumin nanoparticle with arsenic compound loaded with sodium metaarsentie by freeze drying used for the treatment of systemic lupus erythematosus (SLE), rheumatoid arthritis (RA), myasthenia gravis (MG).	[44]

US9,289,499 B2	*Continuous flow production of gelatin nanoparticles (Van Den Broek et al.)* The invention suggests the better way for the production of gelatin based nanoparticles by improvising its reactor to micro reactor in continuous process. This method helps to enhance the productivity, mixing efficiency.	[45]

US8,968,786	*Formation of stable submicron peptide or protein particles by thin film freezing (Johnston et al.)* The present invention focus on the preparation method of micron sized or submicron –sized particles by dissolving in water soluble ingredient with one or more solvents in the vial and by spraying or dripping solvent droplets which is directly exposed to a vapor- liquid interface of less than 500 cm^−1^ area /volume and on surface droplet freezes and convert into a thin film.	[46]

US9,211,283 B2	*Nanoparticle carrier systems based on human serum albumin for photodynamic therapy (Langer et al.)* The invention focus on the formulation of human serum albumin (HAS) nanoparticulate for photodynamic therapy consisting a hydrophobic photosensitizer and a stabilizing agent. This formulation provide abundant therapeutic amount of photosensitizer for parenteral administration. Most widely tetrapyrrole derivatives can be used as photosensitizer as it provides safe and effective formulation of nanoparticles. The prepared formulation is useful in treatment of hyperplasic and neoplasic conditions.	[47]

US8911786 B2	*Nanoparticle comprising rapamycin and albumin as anticancer agent (Desai et al.)* The present invention provide the knowledge the methods, treatment, prevention or delaying cancer by administering nanoparticle consisting rapamycin loaded in protein carrier that is albumin for effective treatment.	[48]

**Table 6:** Various marketed product of protein nanoparticles.

Company	Products	System	Characterization	Currently available products
Emisphere	Eligen®	Carrier molecules	Helps in absorption of small molecules without altering the chemical nature, passive transport helps to cross the membrane	Calcitonin, insulin, PYY, Heparin

Altus	clecr®	Protein crystallization	These catalysts containing the enzyme alcohol dehydrogenase (ADH).	Polypeptides, lipases, proteases.

Abraxis biosciences	Abraxane	Albumin bounded pacitaxel nanospheres	Its active ingredient is paclitaxel and found in injectable suspensions	-
